# Respiratory Diseases

**Published:** 1905-03-18

**Authors:** 


### RESPIRATORY DISEASES.

Tuberculosis.—Wright lays down that we must aim at keeping the protective substances constantly at a higher level and the lymph circulation more active. The agglutination test for this is insufficient, but the phagocytic count shows the richness of the blood in opsonin, one of the most important protective substances. If the increase of the vaccine is too rapid, a negative phase may be caused and actual harm is done. Wright and Douglas[1] have carried out this treatment with some success in obstinate cases of localised tuberculous and staphylococcal infection. Very small doses of vaccine were given every 10 days subject to the opsonic test of the blood each time. Rosenthal[2] advocates respiratory exercises after tuberculous pleurisy to educate the lung, as he believes the early stages of disease are largely due to faults in respiration. Rejecting forced efforts, such as the use of Wolf's bottle, the patient is ordered to breathe so many times to the beat of the hand, then so many diaphragmatic respirations are ordered, and next respirations with the arms held in various positions. These exercises may be continued for months. Osler[3] shows the advantages in tuberculous pleurisy of early aspiration which he thinks is certainly harmless and gives a better chance to the lung to recover. Sweating, purging, and thirst treatment may be useful in a few cases. As soon as the fever has ceased respiratory gymnastics should be employed, such as Wolf's bottle, or Naunyn's chair, and systematic breathing exercises. Samuel West advocates removal of the fluid by a tube reaching to the floor in place of an aspirator. He denies that the lung in these cases becomes bound down by adhesions, though the compressed lung is not indeed protected from tubercle by the delay, and in fact delay may cause infection of the other lung. In tuberculous empyema, the pleura should be irrigated and the flakes washed out. The supposed danger of collapse is not founded on fact. Excision of the rib is undesirable if drainage can be maintained. Acland thinks paracentesis unnecessary in the febrile stage, except it interferes with breathing. In hæmorrhagic effusions, repeated aspiration may be employed. Barr advises early aspiration to be followed by the injection of adrenalin and of sterilised air to the amount of two-thirds of the fluid taken away. He also employs a syphon in place of the aspirator. The injection of the sterilised air prevents distress to the patient and the spread of tuberculous disease in the lung from its engorgement with blood after aspiration.

The Shape of the Chest.—The theory of Woods Hutchinson that the consumptive chest is round instead of flat, and is an undeveloped infantile chest, is examined under the light of fresh data in an exhaustive paper by Lawrason Brown and Pope.[4] They consider that the chest, which is subnormal in its diameters and elongated, is most commonly attacked by tuberculosis, but they confess that they find various inaccuracies in the measurements of the normal chest which they have collected, and these inaccuracies prevent exact conclusions. In health they think the index is about 73, and in the early stages of phthisis this is often reduced, while later on it is increased. However, many cases undergo a change in the direction of flattening, while others become round, but both become reduced in size. This flatness or roundness has no value for prognosis.

Tuberculosis and Heart Disease.—G. W. Norris[5] reviews this much-debated question and contributes the results derived from 1,764 autopsies and numerous clinical records at Philadelphia to the settlement of some of the difficulties. Rokitansky first broached the theory that valvular disease hindered the occurrence of tuberculous disease of the lungs by the congestion it produces, but Frommolt and others have shown this is certainly not in accord with the facts. Another theory was that tuberculosis occurs in persons who have abnormally small hearts. Recent radiographic measurements show that consumptives, except those who have been accidentally infected without predisposition, do have hearts of only half the normal size and that this does not occur in other chronic diseases. The writer finds that these small hearts are very frequent in phthisis, but thinks this is often due to degenerative changes. He acknowledges that congestion from valvular disease should in theory be favourable from the increased transudation into the alveoli, and the muscular and fibrous hyperplasia which takes place. All authors agree that pulmonary stenosis favours the development of phthisis while many insist that mitral stenosis hinders it. However, after reviewing older records and the Philadelphia evidence, Norris concludes that (1) Valvular disease has little, if any, inhibitory influence, and that mitral stenosis does not seem less frequent with phthisis than other heart lesions; (2) while small hearts are frequently found, this hypoplasia may not predispose except by the general under-development which goes with it; (3) pulmonary stenosis does favour the development of phthisis; (4) arterial and endocardial thickening is often produced by tubercular toxins but it is doubtful whether this causes valve lesions; (5) pericarditis, and rarely endo- or myocarditis are produced by the tubercle bacilli, and occasionally we find tuberculosis of the aorta and even a resulting aneurism; (6) fatty fibroid and other changes of the cardiac muscle are commonly produced in phthisis, which explains the frequent failure of cardiac tonics in this disease.

[1] Lancet, Oct. 22. [2] Brit. Med. Jour., Dec. 3. [3] Ibid., Oct. 15. [4] Amer. Jour. Med. Sci., Oct. [5] Ibid.

(*To be continued.*)

